# Detection and analysis of 17 steroid hormones by ultra-high-performance liquid chromatography-electrospray ionization mass spectrometry (UHPLC-MS) in different sex and maturity stages of Antarctic krill (*Euphausia superba Dana*)

**DOI:** 10.1371/journal.pone.0213398

**Published:** 2019-03-11

**Authors:** Xiangning Han, Daicheng Liu

**Affiliations:** Key Laboratory of Animal Resistance, College of Life Science, Shandong Normal University, Shandong, P. R. China; Bahauddin Zakariya University, PAKISTAN

## Abstract

A sensitive and accurate method for determination of 17 endogenous and exogenous steroid hormones in Antarctic krill was developed. The method utilized UHPLC-MS in electrospray ionization mode (ESI). Samples were prepared by alkaline hydrolysis; sequential vortex extraction with ethyl acetate, methanol and acetonitrile; followed by a QuEChERS (Quick, Easy, Cheap, Effective, Rugged and Safe) clean-up method. The system suitability tests including theoretical plate number, resolution, repeatability, tailing factor proved the system’s resolution and reproducibility that can meet the requirements of sample analysis. The developed method resulted in satisfactory recoveries that varied from 75.4%-110.6% and relative standard deviations (RSDs) that ranged from 3.1%-10.5%. The ranges of the limits of detection (LODs) and the limits of quantitation (LOQs) were 2–30 ng kg^-1^ and 10–100 ng kg^-1^, respectively. 14 hormones including cortisone, aldosterone, testosterone propionate, estriol, megestrol acetate, cortisone acetate, dexamethasone, testosterone, hydroxyprogesterone, nandrolone, prednisolone, cortisol, progesterone and estradiol were found in Antarctic krill. Other 3 hormones (Diethylstilbestrol, norethisterone and androsterone) were not detected. The levels of exogenous steroid hormones were much greater than those of endogenous steroid hormones, and the levels of exogenous glucocorticoids were much greater than those of exogenous sex hormones. The changes of hormones in different sex and maturity stages were also explored. Endogenous hormones might regulate the reproductive and development of Antarctic krill. The detected exogenous hormones suggests the potential for hormonal contamination in Antarctic waters that can affect organisms even affect human beings by food chain.

## Introduction

Antarctic krill, *Euphausia superba*, a swarming *Euphausiid* crustacean, is a keystone species of the Antarctic sea ice ecosystem and a commercially valuable species [1]. Antarctic krill contains high quality lipid and protein that can be used in a variety of functional foods [2]. The study of active substances in Antarctic krill has included the measurement of lipids, proteins, and enzymes [3–6]. Studies of crustaceans have shown that the synthesis and metabolism of these substances are regulated by endogenous hormones [7–9]. The physiological activities of Antarctic krill including synthesis of active substances, growth, development, reproduction even swarming may be regulated by hormones similar with other crustaceans, but little is known about endogenous hormones in Antarctic krill. Despite being one of the most abundant species on the planet, there are concerns for the long-term survival of krill stocks given increasing environment contamination, climate change and over-fishing [1, 10]. Diverse profiles and notable concentrations of persistent organic pollutants (POPs), including organochlorine, polychlorinated biphenyl (PCBs), dichlorodiphenyltrichloroethane (DDT), have repeatedly been detected in Antarctic krill [11–13], but to the best of our knowledge, there have been few studies focused on the exogenous hormones contamination in Antarctic krill and Antarctic waters. Therefore, it is of great significance to study endogenous and exogenous steroid hormones in Antarctic krill.

Steroid hormones are vital to normal development, maturation and cell senescence in both vertebrate and invertebrate animals [14, 15]. However, these endogenous and exogenous hormones also pose human health risks in certain circumstances or at excessive amounts [16]. Therefore, monitoring of the abusive use of hormones has become a part of the national surveillance programs of many countries [17,18]. The Codex Alimentarius Commission defines maximum residue limits (MRLs) for several hormonal veterinary drug residues, of which dexamethasone 0.3 μg L^-1^ (milk)-2 μg kg^-1^, ADI value 0–0.015 μg kg^-1^ bw, and for progesterone, testosterone and 17 β-estradiol does not specify MRLs, and their ADI values are 0–30 μg kg^-1^ bw, 0–2 μg kg^-1^ bw, 0–0.05 μg kg^-1^ bw [19]. Nevertheless, illegal use and failure to follow the withdrawal period may lead to high levels of steroid hormones residues in various animal matrices, at levels that may be potentially harmful to consumers [20]. Moreover, these endocrine disrupting compounds (EDCs) show important bioaccumulation effects [21], such that the control of their concentrations in aquatic organisms is absolutely crucial to ensure the quality of seafood. Antarctic krill is directly used as food and has also been developed to produce functional foods in recent years. Krill and krill-derived products are highly consumed, and it is important to study the endogenous and exogenous hormones in Antarctic krill to evaluate the safety of these foods.

Because of the low levels (μg kg^-1^-ng kg^-1^) present in biological samples and the complexity of the food and biological matrices, the analysis of hormones remains a challenging task. An effective sample-preparation process and efficient extraction method are important to achieve optimal sensitivity, selectivity, and specificity. Extraction methods of solvent vortex extraction, matrix solid-phase dispersion, and solvent ultrasonic extraction are commonly used [22, 23]. The solvent vortex extraction method is the simplest, does not require large amounts of solvents, and can be performed rapidly. Most studies have purified hormones from animal samples by solid phase extraction (SPE). SPE requires small volumes of organic solvents, and most impurities are removed during the clean-up, thus avoiding interference with detection [23, 24]. Studies that utilized solvent extraction followed by SPE achieved steroid hormone extraction with high quality and efficiency [23,24].

The development of analytical techniques that allow for extraction and concentration of sex hormones and glucocorticoids from biological tissues at trace concentrations is necessary because the effects of steroids on aquatic biota could be produced even at ng L^-1^ concentrations especially these substances can be bioaccumulated along the food chain. There are many methods to analyze hormones. Gas chromatography with mass spectrometric (GC/MS) detection has been the most commonly used analytical technology for the determination of the steroid hormones [25]. However, this method requires intense purification and a derivation step prior to analysis, thus it is complex and time-consuming [26]. Radioimmunoassay is also a sensitive analytical procedure to detect steroidal hormones in a biological matrix, but its application scope is limited because it cannot be used for all hormones and it is prone to cross activity by endogenous hormones [27]. Importantly, this method can give an inaccurate estimation of steroid levels because the presence of some lipids may interfere with results even after purification [27]. Because Antarctic krill has high levels of lipids, especially phospholipids [2], radioimmunoassay cannot be utilized. Therefore, the development of a more effective and accurate method is of great interest. HPLC is also used as the good way to detect hormones. Ultra-high-performance liquid chromatography-electrospray ionization mass spectrometry (UHPLC-MS) is a promising technique for hormone analysis because of its high selectivity, specificity, and sensitivity. UHPLC became commercially available in 2004. Compared to HPLC, UHPLC uses columns packed with sub-2μm particles at pressures up to 1300 bar, the column length of UHPLC is shorter than HPLC. The particle of UHPLC column is smaller than the HPLC column and the pressure is higher. Because of the high sensitivity, a significantly diminished solvent sample consumption and the shorter analysis time, UHPLC is often used for the detection of trace substances in complex matrices [28]. UHPLC-MS is rapid and does not require derivatization steps. Importantly, this method is sensitive, providing limits of detection (LODs) and limits of quantitation (LOQs) that are sufficiently low to allow for the analysis of a wide range of steroidal hormones, including trace hormones [29, 30]. However, to the best of our knowledge, applications of UHPLC-MS to animal-derived foods have been limited to reports of steroid hormones in beef, plasma, and a handful of aquatic biology (most are fish samples). Rayco Guedes-Alonso et al. detected 15 steroid hormones in fish tissues, which were detected by microwave-assisted extraction and detected by ultra-high-performance liquid chromatography-mass spectrometry. The established method has good sensitivity, suitable detection limit and higher than 50% recovery rate. The concentration of eight detected compounds ranged from below the quantification limits to 3.95 μg g^-1^ [21]. Anna Jakimska et al. detected 19 endocrine disruptors in several kinds of fishes purified by QuEChERS and then detected by UHPLC-MS, including 7 hormone substances. Seven substances were all detected in fishes. The recovery rates ranged from 40% to 103% for most substances, and the detection limits ranged from 0.002 to 3.09 ng g^-1^. The accuracy was less than 20% [31]. But there were no related researches in Antarctic krill. Thus, it is really important to construct a rapid and sensitive method to detect hormones in Antarctic krill and krill products.

In this study, a reliable and sensitive UHPLC-MS analytical method was constructed and used to detect 17 endogenous and exogenous steroid hormones including estrogens, androgens, progestins, glucocorticoids, and mineralocorticoids in Antarctic krill. The instrument conditions and mobile phases were optimized. The krill samples were extracted based on alkaline hydrolysis; ethyl acetate, methanol and acetonitrile vortex extraction respectively, followed by a clean-up step with QuEChERS method. Conditions with and without enzyme hydrolysis were also compared, and use of HLB and C_18_ solid-phase extraction cartridges for sample preparation were also tested. The extraction and purification methods were optimized, and used to analyze Antarctic krill samples. Fourteen hormones were detected and three other hormones were not detected. This method was applied to analyze the levels of hormones for Antarctic krill of different sex and maturity stages.

## Materials and methods

### Instrumentation

The vortex mixer (MX-S) was purchased from Scilogex Co. (USA). The high-speed freezing centrifuge (5810 R) was obtained from Eppendorf Co. (Hamburg, Germany). The Eyela rotary evaporator (N-1100) was obtained from RIKAKIKAI Co. (Tokyo, Japan). Thermo UltiMate^™^ 3000 was purchased from Thermo Fisher Scientific Co. (New York, USA). The TSQ Vantage was obtained from Thermo Fisher Scientific Co. (New York, USA). The freeze dryer (Biosafer-10 B) was purchased from Biosafer Co. (Nanjing, CN).

### Samples, chemicals, reagents, and materials

Frozen whole Antarctic krill were purchased from the Liaoning Fishery Group (Dalian, Liaoning, China). The batch of Antarctic krill used in this study (35–55 mm in body length) was caught in the first quarter of 2017 from the waters surrounding the Chinese Great Wall Antarctic Station (48.1–48.3 zone in the Antarctic). Standard preparations of progesterone, androsterone, testosterone, estradiol, estriol, aldosterone, cortisol, cortisone, hydroxyprogesterone, nandrolone, diethylstilbestrol, norethisterone, testosterone propionate, megestrol acetate, prednisolone, cortisone acetate, and dexamethasone (all at a purity ≥ 98%) were obtained from Sigma-Aldrich (St. Louis, MO, USA) and were stored at -20°C. The molecular structures of all standard were shown in S1 Fig. HPLC grade solvents of methanol and acetonitrile were purchased from Fisher Scientific International Inc. (Pittsburgh, USA). Methanol, formic acid, acetonitrile, sodium hydroxide, glacial acetic acid, and sodium acetate were analytical-grade reagents (AR). β-glucuronidase was purchased from Sigma-Aldrich (St. Louis, MO, USA). HLB and C_18_ solid phase extraction cartridges, each containing 60 mg material (3 mL), were purchased from Waters Co. (Milford, MA, USA). Amino-propyl solid phase extraction cartridges, each containing 200 mg material (3 mL), were purchased from Waters Co. (Milford, MA, USA). QuEChERS solid phase extraction cartridges (2 mL) were obtained from Copure Co. (Shenzhen, CN).

Stock solutions were prepared for all standard substances at 1000 mg L^-1^ in methanol. Concentration gradients of the standard solutions were obtained by diluting the stock solutions with methanol. The prepared standard solutions were stored at—4°C.

### Sample preparation

This study was approved by the Shandong Institute of Zoology and followed all applicable international, national and/or institutional guidelines for the care and use of animals. Krill samples were cut from the middle section of a chunk of frozen krill to avoid any contamination with hormones introduced from packaging or transportation. First, 10 g of fresh Antarctic krill was crushed and ground. The sample was then extracted three times with 10 mL of ethyl acetate by vortex extraction. The supernatants were combined and transferred to a 50 mL centrifuge tube. To the remaining residue in the tube, 5 mL sodium hydroxide buffer (pH = 12) was added and then vortexed for 1 min. The mixture was then centrifuged at 4000 rpm for 5 min at 4°C. The supernatant was then transferred to the 50 mL centrifuge tube described above. The combined supernatant was then evaporated to dryness (50°C) and then reconstituted with ethyl acetate (1 mL). The solution was filtered by 0.22 μm organic filter membrane. Methanol and acetonitrile were also used as the extraction solvents, the procedure was repeated as above mentioned.

For clean-up, the above-prepared solutions were transferred into a 2 mL QuEChERS SPE cartridge already containing 25 mg PSA, 50 mg GCB and 150 mg anhydrous MgSO_4_. The tube was closed, vortexed for 2 min, and then centrifuged for 5 min at 4000 rpm. The supernatant was dried under a gentle nitrogen stream and reconstituted with extraction solvents (1 mL) for UHPLC-MS analysis. The procedure for the extraction, clean-up of steroid hormones in Antarctic krill sample was shown in S2 Fig.

The SPE column, filter, and pestle were all prewashed with ultra-pure water, followed by methanol. All glassware were cleaned and then heated for 3 h at 400°C. In addition, procedural blanks were included in each batch of samples to ensure minimal contamination.

### UHPLC-MS analysis

The analysis was done on a Thermo UltiMate^™^ 3000 coupled with a Thermo Scientific TSQ Vantage. An Acchrom Unitary C_18_ column (2.1 mm×150 mm, 5 μm) was used for separation. The column oven was maintained at 30°C, and the injection volume was 10 μL. Water containing 0.1% formic acid (A) and Methanol (B) were used as the mobile phase with a total flow of 0.2 mL min^-1^. Gradient elution was performed as follows: methanol was decreased from 80% to 70% in 8 min and then decreased from 70% to 65% in 2 min.

The mass spectrometer was operated in both positive and negative electrospray ionization mode (ESI) with multiple-reaction monitoring (MRM). The capillary temperature was 350°C. The vaporization temperature was 300°C. The sheath gas pressure was set to 35 Arb, the auxiliary gas pressure was set to 20 Arb, the spray voltage of ESI^+^ was set to 3.5 KV, and the spray voltage of ESI^-^ was set to 3.0 KV. For each hormone, both the parent and product ions were selected and the collision energy was optimized for maximum intensity. The relative parameters were shown in S1 Table. The gradients of standard solutions (1, 5, 10, 20, 50, 100, 200, and 500 μg kg^-1^) were analyzed by UHPLC-MS to determine the linearity of the detection.

### System suitability test

**Fig 1:**
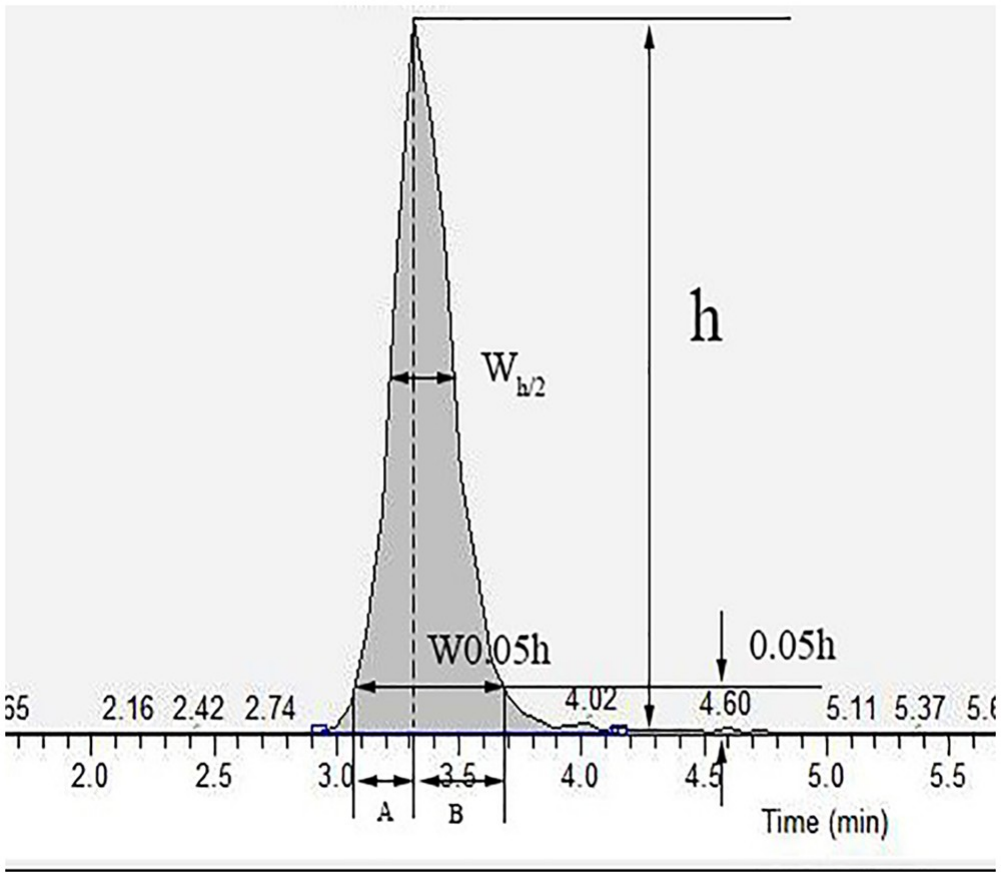
The calculation indexes about tail factor. The W0.05h is the 0.05h peak width, and the A was the width of left half peak, and the B was the width of right half peak.

Chromatographic system suitability test usually includes theoretical plate number, resolution, repeatability, tailing factor. The first complex system suitability test giving information on a large variety of performance parameters of capillary columns was developed by Grob et al [32]. We did the system suitability test in this research according to the standard. The calculation of performance parameters are as following:

1. Theoretical plate number of chromatographic column
  The theoretical plate number of using column with the setting conditions on the basis of 17 kinds of standard substances. The calculation formula:
  $$n=5.54{\left(\frac{t_{R}}{W_{1/2}}\right)}^{2}$$ (1)
  Where *n* is theoretical plate number, *t*_*R*_ is the retention time, and the *W*_*1/2*_ is the half peak width.
2. Resolution
  Resolution is the degree of separation about the main substance peak and the impurity peak. The calculation formula is as follows:
  $$R=2\frac{\left(t_{R2}-t_{R}{}_{l}\right)}{W_{l}+W_{2}}$$ (2)
  Where *R* is the resolution, *t*_*R1*_ is the retention time of the peak 1, *t*_*R2*_ is the retention time of peak 2, *W*_*1*_ is the peak width of peak 1, *W*_*2*_ is the peak width of peak 2.
3. Repeatability
  The resolution is obtained by repeating the injection 5 times and calculating the peak area. The relative standard deviation of the peak area measurement should be no more than 2.0%.
4. The tail factor.
  To guarantee the separation effect and measurement accuracy, the tail factor should be checked. The Fig 1 showed the indexes about calculation tail factor. The calculation method was on the basis of the formula:
  $$T=\frac{W0.05h}{2A}=\frac{\left(A+B\right)}{2A}$$ (3)
  Where the *W0*.*05h* is the 0.05h peak width, and the *A* was the width of left half peak, and the *B* was the width of right half peak.

### The recovery method and real sample analysis

The recovery was evaluated using 10 g samples spiked with three gradients of standard analyte. The precision was expressed as percentages of RSDs, and was determined for each hormone in the spiked samples. The LOD and LOQ for each compound were calculated by determining the signal-to-noise (S/N) ratio of the lowest measured concentration and extrapolating to S/N ratios of 3 and 10, respectively for the diagnostic transition ions. The proposed method was applied to the analyses of the 17 steroid hormones in Antarctic krill (n = 3). For each hormone, the content was calculated as follows:

$$Q\left(ng\;g^{-1}\right)=\frac{A\times 1000\;\mu L}{10\;\mu L\times 10\;g}\left(ng\;g^{-1}\right)$$ (4)

*Q* represents the hormone content in Antarctic krill, *A (ng)* was the amount of hormones in *10 μL* sample, *1000 μL* was used as the sample volume, *10 g* was the weight of fresh Antarctic krill sampled, and *10 μL* was the sample volume.

### Hormone analysis in Antarctic krill at different sex and maturity stages

The Antarctic krill individuals were divided into different sex (female and male) and different maturity stages. The sex was determined by measuring the length and width of the carapace [33]. The maturity stages (six stages according to body length: 0 + (6.7 ± 1.2 mm), 1+ (28.7 ± 3 mm), 2 + (36.0 ± 2.9 mm), 3 + (43.6 ± 3.3 mm), 4 + (50.3 ± 1.9 mm), 5 + (54.2 ± 2.2 mm) were determined by measuring the body length [34]. Of our specimens, no krill of stage “0” were present. So, we divided the krill from “1” stage. The amounts of hormones were determined by UHPLC-MS (n = 3), and the differences for different sex and maturity stages were analyzed.

## Results and discussion

### Optimization of UHPLC-MS

Ionization ESI sources occur in the solution state, so the composition of the mobile phase has a significant influence on the response of the solute. Thus, effective separation of compounds requires optimization of the mobile phase gradients. We first tested methanol and acetonitrile, and observed peaks for all hormones within 3 min that could not be completely separated. Next, methanol and water with 0.1% formic acid was used as the mobile phase, and we obtained complete separation of the 17 steroid hormones. We used this formulation of the mobile phase for subsequent experiments.

A higher flow rate corresponds to a lower ionization efficiency, we observed good sensitivity in a range between 0.1 mL min^-1^–0.3 mL min^-1^. Flow rate should be tailored to the selected column. To balance separation efficiency and ionization efficiency, the flow rate was finally set at 0.2 mL min^-1^.

**Fig 2:**
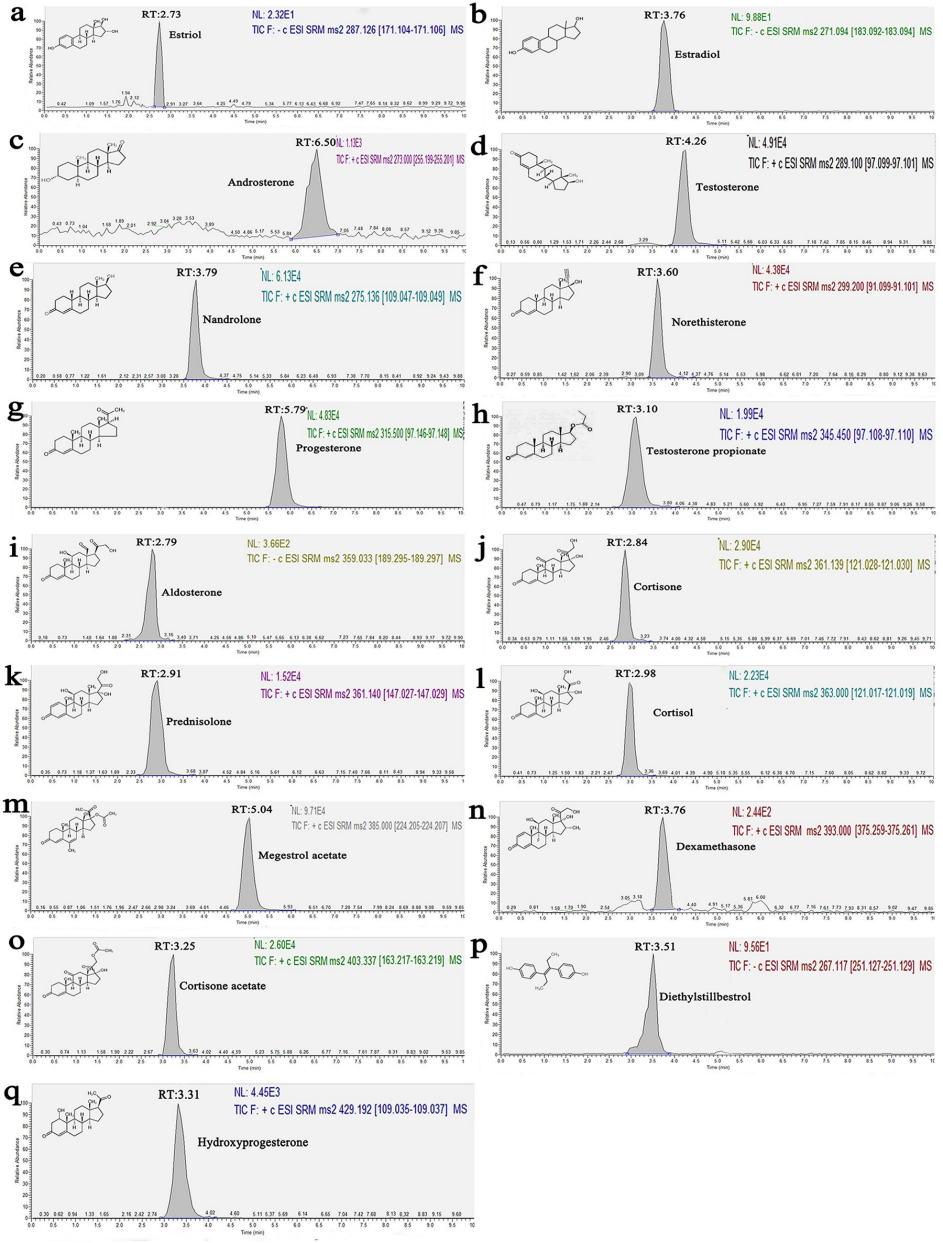
UHPLC-MS chromatograms of standard solution.

For the mass spectrometry, negative ionic mode (ESI^-^) detected estrogen, and positive ion mode Ionization (ESI^+^) detected progesterone, androgens, glucocorticoids, and mineralocorticoids. The chromatograms of these hormones are shown in Fig 2. The peaks of all the hormones appeared in 9 min, and all the hormones were completely separated, suggesting that these conditions were feasible for this analysis. The linearity of detection for each hormone, linear range, LODs, and LOQs are listed in Table 1. The LODs ranged from 2–30 ng kg^-1^ and LOQs ranged from 10–100 ng kg^-1^. Compared with previously published methods [24,34], the LODs of this method were approximately the same or less. This ensures the reliable determination of hormones at levels lower than the national minimum required performance limits (2–50 μg kg^-1^ for the xenohormones) of EU [35] and the US [36] residue limits for veterinary drugs (0.12–3 μg kg^-1^ for natural hormones). Overall, the results indicated that the method could be used to monitor the trace residues of steroid hormones in krill sample.

**Table 1:** The linear equation, linear range, LODs and LOQs.

Compound	Linear equation	R^2^	Linear range (μg L^-1^)	LOD ng kg^-1^	LOQ ng kg^-1^
Cortisone	Y = -1411.73+467.79×X	0.9974	10~500	30	100
Aldosterone	Y = -68.43+5.43×X	0.9972	10–500	30	100
Testosterone Propionate	Y = -4301.17+352.61×X	0.9971	10–300	30	100
Estriol	Y = -2.64+0.77×X	0.9927	1–250	2	10
Megestrol acetate	Y = -10868.6+3651.34×X	0.9922	1–500	2	10
Cortisone acetate	Y = -897.301+295.598×X	0.9929	1–500	2	10
Dexamethasone	Y = 2188.64+77.82×X	0.9942	5–500	10	50
Testosterone	Y = -9915.72+975.45×X	0.9953	1–500	2	10
Hydroxyprogesterone	Y = 270.23+76.56×X	0.9911	10–500	30	100
Diethylstilbestrol	Y = -60.25+4.18×X	0.9909	10–500	2	10
Nandrolone	Y = -11696.1+1094.08×X	0.9903	1–500	2	10
Prednisolone	Y = -663.42+280.98×X	0.9977	1–500	2	10
Cortisol	Y = -1117.15+370.51×X	0.9941	1–500	30	100
Norethisterone	Y = -3118.5+718.62×X	0.9929	1–500	30	100
Androsterone	Y = 8.88+33.03×X	0.9925	10–500	30	100
Progesterone	Y = -10985.1+1055.84×X	0.9931	10–500	30	100
Estradiol	Y = -3.05+0.51×X	0.9944	10–500	30	100

### The system suitability test results

The results of theoretical plate number, resolution, repeatability, tailing factor were shown in the Table 2. The theoretical plate number based on each substance were all high than 5000 which are met the requirements of liquid chromatography. The resolution was not very high. However, under the conditions of mass spectrometry, each peak has a good peak shape and can be accurately qualified and quantified. Due to the complexity of the food matrix, the resolution can meet the requirements. The repeatability was really good that all the SD values are lower than 2.0%. Finally, the tailing factor of each substance was lower than 1.2 and higher than 0.9 which don’t influence quantification by mass spectrometry. The system suitability test results approved that the system’s resolution and reproducibility that can meet the requirements of hormones analysis in Antarctic krill.

**Table 2:** The theoretical plate number, resolution, repeatability, tailing factor results.

	**Prednisolone**	**Megestrol acetate**	**Dexamethasone**	**Cortisone acetate**	**Hydroxyprogesterone**	**Diethylstillbestrol**
**Theoretical plate number**	6438.8	8654.2	6617.8	6821.8	6632.1	5828.7
**Average peak areas±SD**	54729.8±0.769	346369.4±0.917	28830.6±1.398	33252.2±1.599	44405.2±1.460	1571.0±1.198
**Resolution**	1.83	1.41	1.93	1.10	1.80	ND
**The tail factor**	1.05	1.1	0.98	1.1	1.08	0.90
**Continued:**						
	**Nandrolone**	**Norethisterone**	**Progesterone**	**Testosterone propionate**	**Cortisone**	**Cortisol**
**Theoretical plate number**	6221.5	6482.6	8321.4	6328.1	6832.6	6651.2
**Average peak areas±SD**	229107.2±0.173	43167.8±1.549	93432.4±1.549	122335.8±1.772	37132.0±1.984	87319.6±1.830
**Resolution**	1.36	ND	1.18	1.60	1.75	1.34
**The tail factor**	1.1	1.1	1.05	1.05	0.99	1.01
**Continued:**						
	**Aldosterone**	**Androsterone**	**Testosterone**	**Estradiol**	**Estriol**	
**Theoretical plate number**	6745.9	6432.8	7865.9	7634.8	6242.4	
**Average peak areas±SD**	5247.8±0.426	11900.8±0.781	388847.4±0.644	1486.2±1.228	1910.2±0.678	
**Resolution**	1.94	ND	1.60	1.56	1.13	
**The tail factor**	0.90	0.92	1.05	0.92	1	

### Optimization of sample preparation

**Fig 3:**
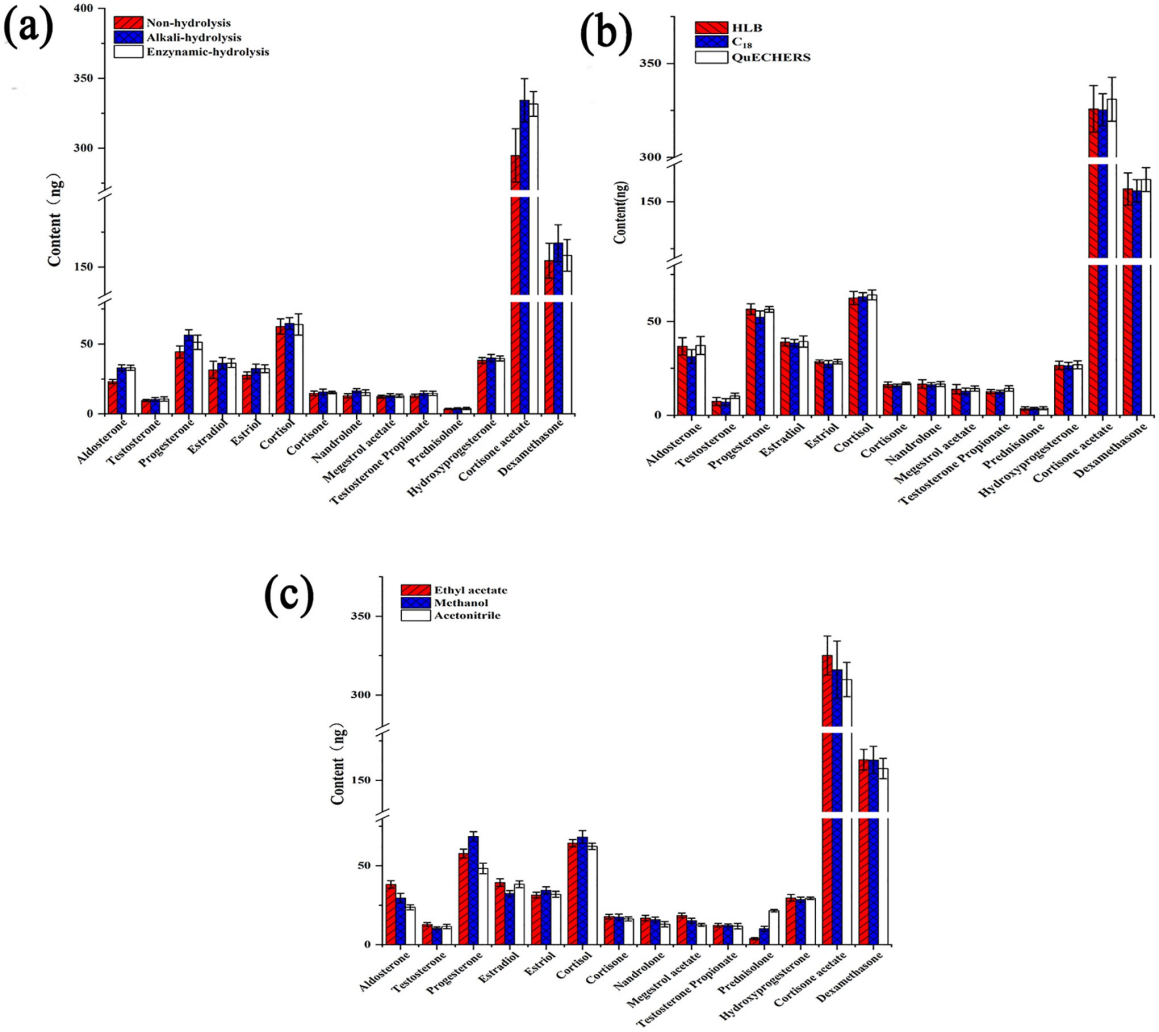
The results of optimization. a shows the effect of hydrolysis. b shows the effect of purification. c shows the effect of extraction solvents.

Previously published protocols differ in the use of enzymatic or alkaline treatment for a hydrolysis step in sample preparation. In some protocols, this step is required [22, 24]. However, some recent papers have shown that omitting a hydrolysis step has no significant effect on the efficiency of extraction of hormones [22,24]. Here, we tested enzymatic hydrolysis, alkaline hydrolysis, or no hydrolysis during sample preparation. Enzymatic hydrolysis was performed by grinding freeze-dried krill (10 g of fresh krill freeze-dried at—40°C) to powder. Next, 12 mL of 2 mol L^-1^ acetate buffer (pH 5.2) was added, followed by the addition of 100 μL of β-glucuronidase and then overnight incubation at 37°C. The sample was cooled to room temperature and then extracted without alkaline hydrolysis. The no-hydrolysis condition was also tested by solvent vortex extraction. All procedures were repeated 3 times, and then the amount of hormones in 10 μL of each sample was determined. The effects of three methods are shown in Fig 3(a). Aldosterone, testosterone, and estradiol were effectively extracted by enzymatic hydrolysis, but for other hormones, alkaline hydrolysis was better than enzymatic hydrolysis. For the majority of hormones, extractions were not sufficient in the absence of hydrolysis. Although enzymatic hydrolysis worked for some hormones, the requirement of complex pretreatment and the high cost of enzymatic hydrolysis made this a less attractive option. Thus, alkaline hydrolysis was selected as a simple and effective hydrolysis method.

To determine the best solvents to extract steroid hormones in Antarctic krill, ethyl acetate, methanol, and acetonitrile were tested. The procedure was as described in the sample preparation section, and the results ae shown in Fig 3(b). Interestingly, different hormones were extracted more efficiently in different solvents. Ethyl acetate was better for aldosterone, testosterone, cortisone, nandrolone, testosterone propionate, megestrol acetate, hydroxyprogesterone, cortisone acetate, and dexamethasone extraction. Progesterone, estradiol, estriol and cortisol were effectively extracted by methanol. Acetonitrile showed the best extraction efficiency for prednisolone than other solvents. Due to variation in the efficiency of extraction for the different solvents, all three solvents were used to extract hormones in Antarctic krill.

SPE is the most common extraction method used for the isolation of hormones from an animal or food matrix, and provides excellent extraction efficiency. To develop a fast and simple method for the treatment of samples of Antarctic krill, HLB SPE cartridge, C_18_ SPE cartridge, and QuEChERS method were tested for sample preparation. For the HLB cartridge, 6 mL water and 6 mL methanol were separately used to flush the column before application of the sample to the column and then flushing with 6 mL water. The amino-propyl solid phase extraction cartridge was followed by the HLB column cooperated with HLB column. Then, 8 mL of methanol was used to flush and the eluent was concentrated to 1 mL. The C_18_ cartridge was used like the HLB column. The QuEChERS method was performed as described in the sample preparation section. The results are shown in Fig 3(c). We can see that use of HLB was able to extract progesterone, estradiol, and nandrolone in Antarctic krill. C_18_ did not allow extraction for most hormones, possibly due to the matrix adsorbent effect of the C_18_. The QuEChERS method has good effects on the extraction of most of the Antarctic krill hormones. Because the QuEChERS method is simple, fast, and uses little solvent, this method was chosen for the hormone extraction method.

Thus, the optimal extraction parameters were determined as alkaline hydrolysis; sequential ethyl acetate, methanol, and acetonitrile vortex extraction; and a clean-up step with QuEChERS method.

### The results of method recovery and the real sample analysis

**Fig 4:**
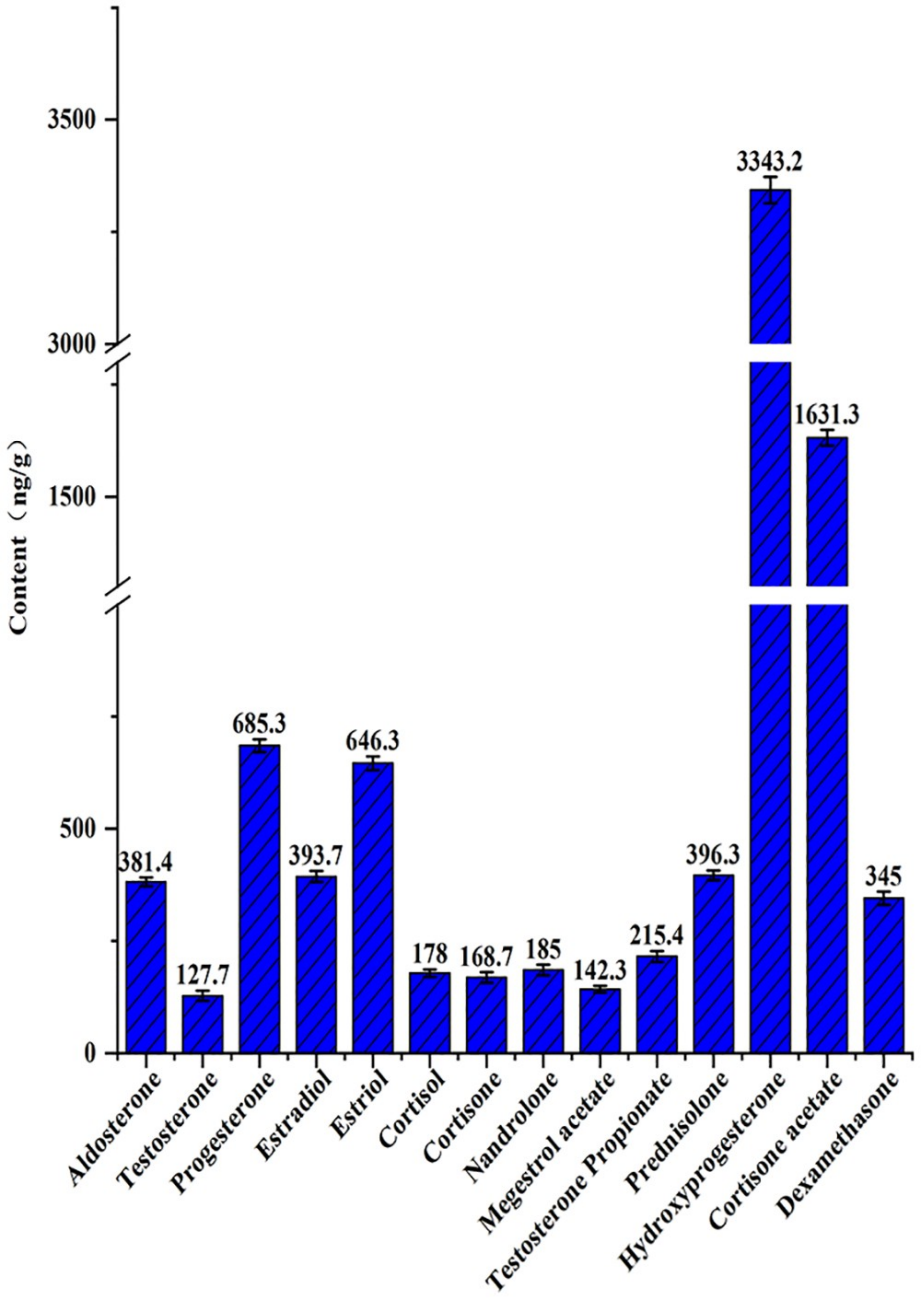
Detected hormones and their contents in Antarctic krill.

The recovery and relative RSD of the method are listed in Table 3. The results gave the recovery at three spiking levels ranging from 75.4% to 110.6%, and RSDs that ranged from 3.1% to 10.5%. The average recovery was higher than 70%. Reproducibility, as represented by RSD percentages, was within 11%, which is very satisfactory. A real sample survey was conducted to detect hormones in Antarctic krill samples, as shown in Fig 4. Diethylstilbestrol, androsterone and norethindrone were not detected in Antarctic krill. The aldosterone, testosterone, progesterone, estradiol, estriol were believed to be produced by crustaceans themselves (Endogenous steroid hormone). These hormones are found in Antarctic krill. Other steroid hormones are synthetic hormones (Exogenous steroid hormones). Contents of hormones: cortisone acetate > dexamethasone > cortisol > progesterone > hydroxyprogesterone > estradiol > aldosterone > estriol > prednisolone > testosterone propionate > cortisone > nandrolone > megestrol acetate > testosterone. The levels of exogenous hormones were significantly greater than those of endogenous hormones, and the levels of exogenous glucocorticoids were higher than those of exogenous sex hormones. The detection of exogenous hormones showed potential hormone contamination of Antarctic waters, especially with glucocorticoids. The results suggest the need to more closely examine Antarctic environment contamination. Antarctica is a relatively closed place, but in the last few decades, the amount of chemicals released into the environment has increased considerably. Among these compounds, hormone residues are a cause for concern because they can affect the biological activity of non-target organisms [24]. These compounds present a potential risk for wildlife and humans through the consumption of contaminated food. Hormones influence the endocrine system, and they will disrupt the physiologic function of other active substances, and even influence the sexual development [16]. Excessive hormones can lead to endocrine disorders in the human body and contribute to diseases such as cancer. Thus, the finding of hormones in Antarctic krill has important implications for the safety of Antarctic krill and Antarctic krill products.

**Table 3:** Recovery and relative standard deviation (RSD) of different kinds of hormones.

Compound	Concentration (μg kg^-1^)	Recovery (%)	RSD (%)
Cortisone	10, 15, 20	89.3, 91.6, 88.8	4.5, 5.8, 5.6
Aldosterone	10, 15, 20	94.6, 98.9, 92.1	9.1, 7.5, 6.8
Testosterone Propionate	10, 15, 20	101.4, 100.1, 110.6	6.3, 6.8, 7.9
Estriol	1, 5, 10	75.4, 82.1, 87.5	5.3, 4.9, 5.8
Megestrol acetate	1, 5, 10	92.4, 95.9, 93.8	6.7, 6.3, 6
Cortisone acetate	1, 5, 10	82.4, 85.6, 81	3.5, 4.2, 5
Dexamethasone	5, 10, 15	79.7, 76.1, 84.5	8.1, 7.5, 7.2
Testosterone	1, 5, 10	91.2, 94.5, 99.6	9.2, 9.6, 9
Hydroxyprogesterone	10, 15, 20	101.1, 102.9, 107.3	10.5, 7.3, 8.2
Nandrolone	1, 5, 10	88.4, 81.2, 89.6	9.5, 6.8, 4.8
Prednisolone	1, 5, 10	95.2, 93.6, 99.9	6.2, 5.3, 3.9
Cortisol	1, 5, 10	104.4, 108.1, 102.2	7.8, 8.4, 9.1
Progesterone	10, 15, 20	94.1, 96.3, 95.2	4.2, 4.9, 3.1
Estradiol	10, 15, 20	84.3, 89.5, 94.2	10.4, 9.5, 9.2
Diethylstilbestrol	10, 15, 20	82.4, 83.6, 85.2	4.6, 5.2, 4.9
Norethisterone	10, 15, 20	92.1, 95.6, 93.2	5.3, 4.6, 5.2
Androsterone	1, 5, 10	88.6, 87.4, 89.2	4.8, 5.3, 6.1

### Changes of hormones in different sex and maturity stages in Antarctic krill

**Fig 5:**
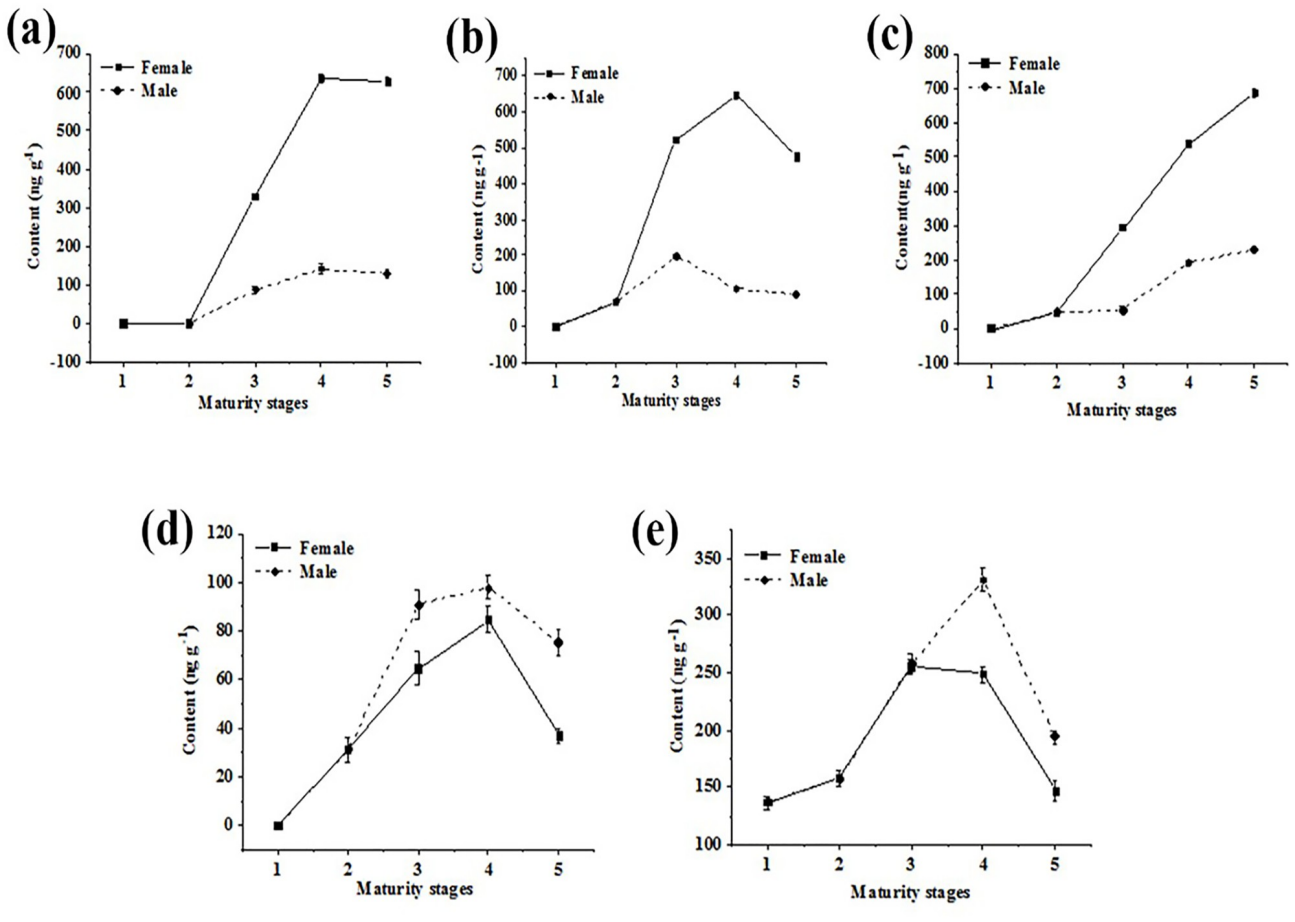
The changes of endogenous steroid hormones. Graph a, b, c, d and e show the trends of progesterone, estradiol, estriol, testosterone, and aldosterone, respectively.

**Fig 6:**
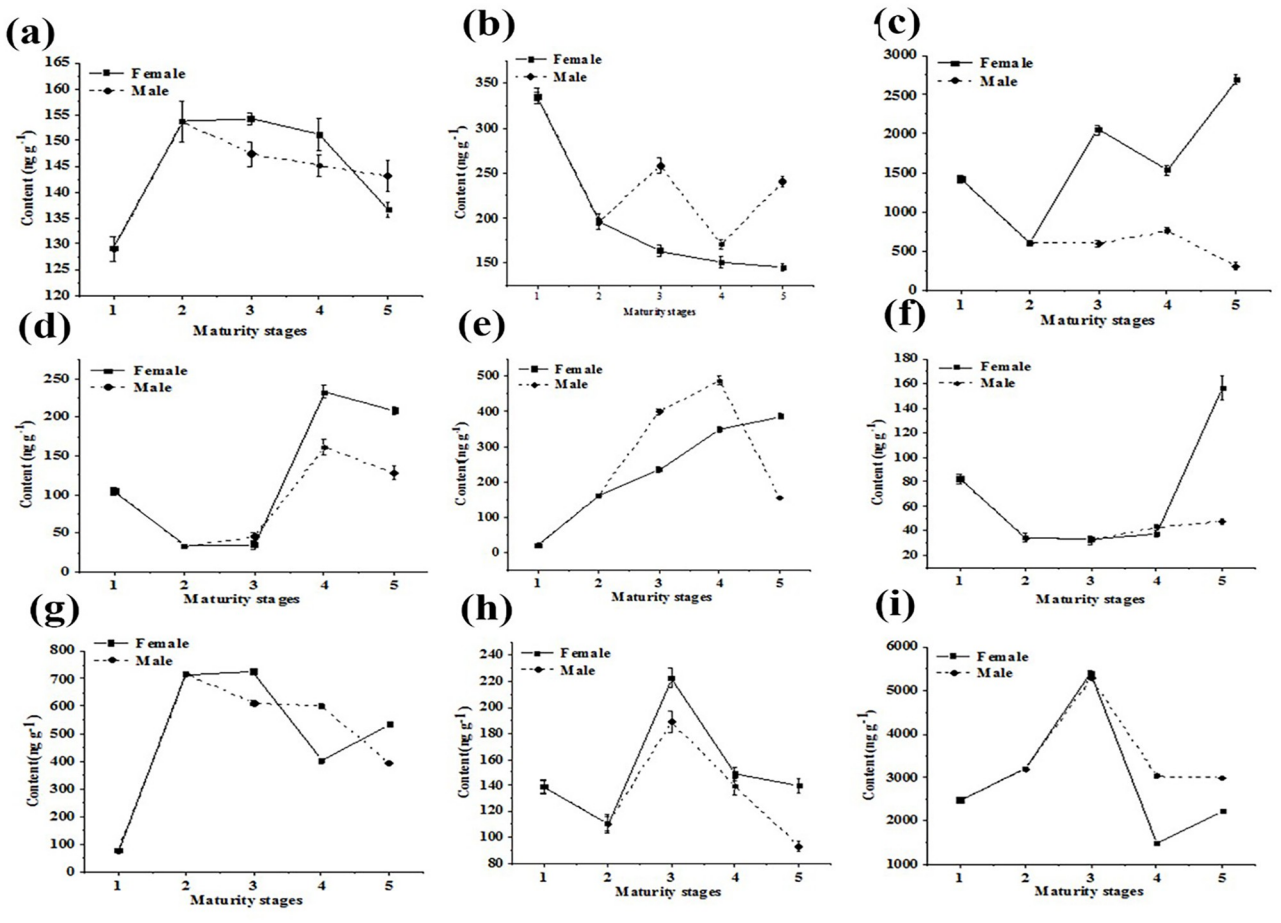
The changes of exogenous steroid hormones. Graphs a, b, c, d, e, f, g, h, and i show testosterone propionate, nandrolone, dexamethasone, prednisolone, hydroxyprogesterone, megestrol acetate, cortisol, cortisone, and cortisone acetate, respectively.

The contents of hormones were compared for different sex and maturity stages in Antarctic krill, as shown in Figs 5 and 6 shows the changes of endogenous steroid hormones. Progesterone, estradiol, estriol, and testosterone were present at significantly different levels in female and male krill. Their levels increase during maturation and then decrease once fully mature. This result is similar to some reports about other crustaceans [37–40]. These changing levels of hormones may be related to the development and reproduction of Antarctic krill, but the detailed mechanisms require further study. Aldosterone was present at a higher level in males, suggesting differences in the water and salt balance between male and female krill. However, this should be tested further. The contents of aldosterone were highest at the 4 stage, the same trend as the endogenous sex hormones. This may suggest a role for aldosterone to regulate sex hormones, but this also requires further investigation. The levels of exogenous androgens are shown in Fig 6. Testosterone propionate is an androgen hormone, but was present at a high level in females in stages 3 and 4. This may be related to the high lipid content and accumulation in the female individual, or reflect differences in absorption, utilization, and decomposition. In the 5 stage of the female individual, the content of megestrol acetate was extremely high. The contents of prednisolone, dexamethasone and cortisone were higher in females than males, suggesting that these hormones may accumulate in high lipid female individuals. The contents of nandrolone and cortisone acetate were higher in males, and the nandrolone level decreased from 1 to 5 stages in females, perhaps indicating better utilization, decomposition, or elimination in females. The trends of cortisone and cortisone acetate are similar, consistent with similar structures, but the overall amounts were quite different. This may reflect the widespread clinical use of cortisone acetate, resulting in high environmental contamination. The overall content of hydroxyprogesterone in males was also greater than in the female individual, but the content was constantly rising in the female individual. This might suggest better utilization and accumulation in females. Overall, it is important to determine if exogenous steroid hormones interfere with the physiological activities of Antarctic krill or interfere with the secretion of endogenous hormones.

## Conclusions

A UHPLC-MS method was developed to measure 17 steroid hormones in Antarctic krill samples. The conditions for UHPLC-MS and sample preparation were optimized. Samples were prepared using alkaline hydrolysis; sequential ethyl acetate, methanol, and acetonitrile vortex extraction; and a QuEChERS clean-up step. The observed good linearity; recoveries; and satisfied RSD, LODs, and LOQs all meet the demands of hormone analysis. Samples from real Antarctic krill were used. Diethylstilbestrol, Androsterone, and Norethindrone were not detected in Antarctic krill, but 14 other hormones were found. There were differences in hormone levels for samples of different sex and maturity stages. Endogenous steroid hormones showed significant differences for different sex and maturity stages, suggesting these hormones may regulate the reproduction and development of Antarctic krill. The detection of exogenous steroid hormones should serve as an alert to the risk of hormonal contamination of the Antarctic waters and has important implications for the need to assure the safety of Antarctic krill and Antarctic krill product.

## Supporting information

S1 Fig Molecular structure of the 17 kinds of steroidal hormones. (TIF)

S2 Fig Analytical procedure for the extraction, clean-up of steroid hormones in Antarctic krill sample. (TIF)

S1 Table MS acquisition parameters at the ionization model of positive (ESI+) and negative (ESI-). (DOCX)

S2 Table The data of optimization. The effects of hydrolysis, purification and extraction solvents.
(DOCX)

S3 Table Detected hormones and their contents in Antarctic krill. (DOCX)

S4 Table The changes of endogenous steroid hormones and exogenous steroid hormones. (DOCX)

## Abbreviations

AR: analytical-grade reagents
DDT: dichlorodiphenyltrichloroethane
ESI: electrospray ionization mode
EU: European Union
GC/MS: gas chromatography with mass spectrometric
LODs: limits of detection
LOQs: limits of quantitation
MRM: multiple-reaction monitoring
PCBs: polychlorinated biphenyl
POPs: persistent organic pollutants
RSDs: relative standard deviations
S/N: signal-to-noise
SPE: solid phase extraction
UHPLC-MS: ultra-high-performance liquid chromatography-electrospray ionization mass spectrometry
US: the United States
