# Supplementary figures and images for: Detection and analysis of 17 steroid hormones by ultra-high-performance liquid chromatography-electrospray ionization mass spectrometry (UHPLC-MS) in different sex and maturity stages of Antarctic krill (Euphausia superba Dana)
Source: PLoS One. 2019 Mar 11;14(3):e0213398. doi: 10.1371/journal.pone.0213398 (PMC6411355; doi:10.1371/journal.pone.0213398)

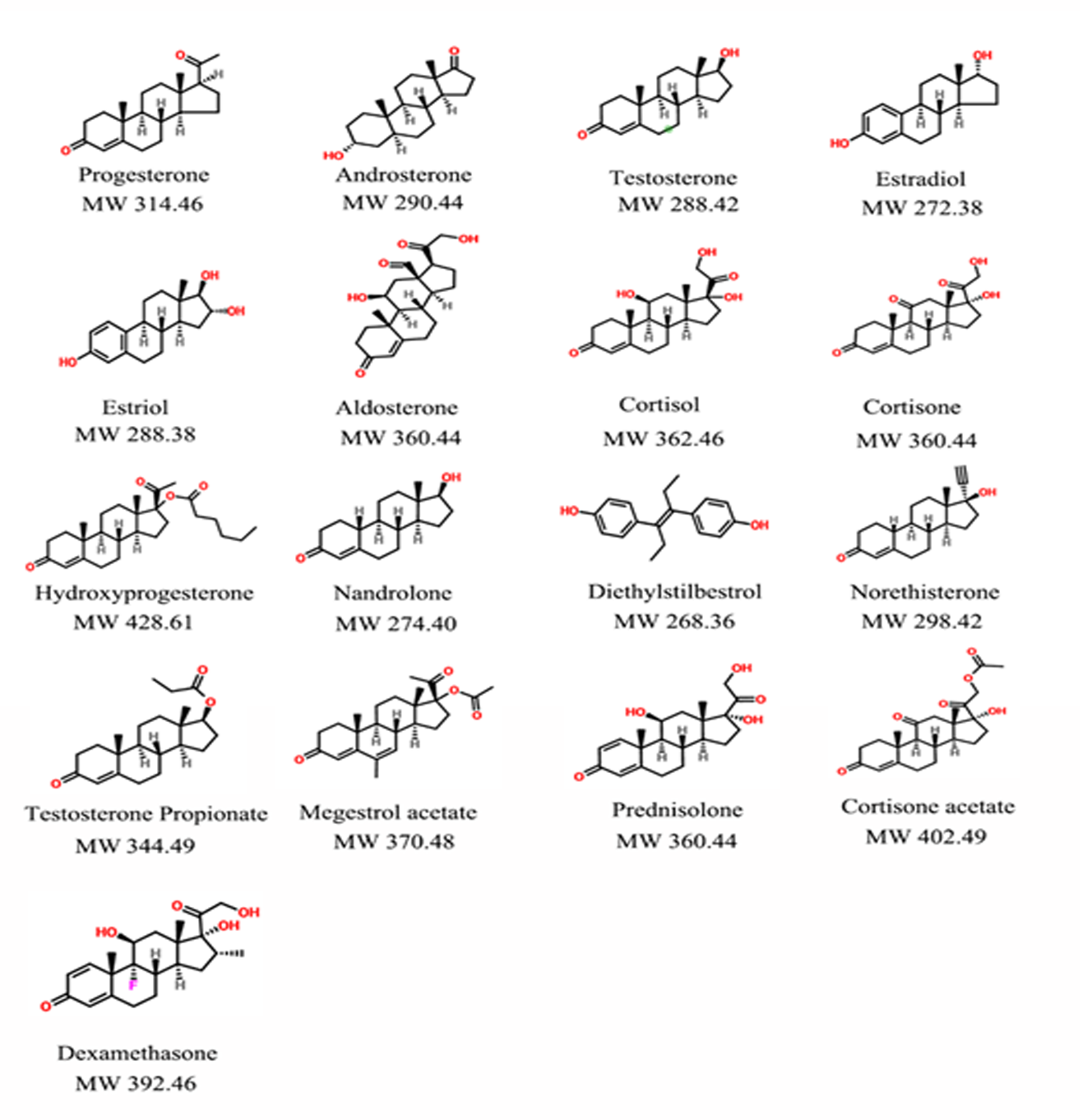

Supplement: S1 Fig — (TIF) [file pone.0213398.s001.tif]

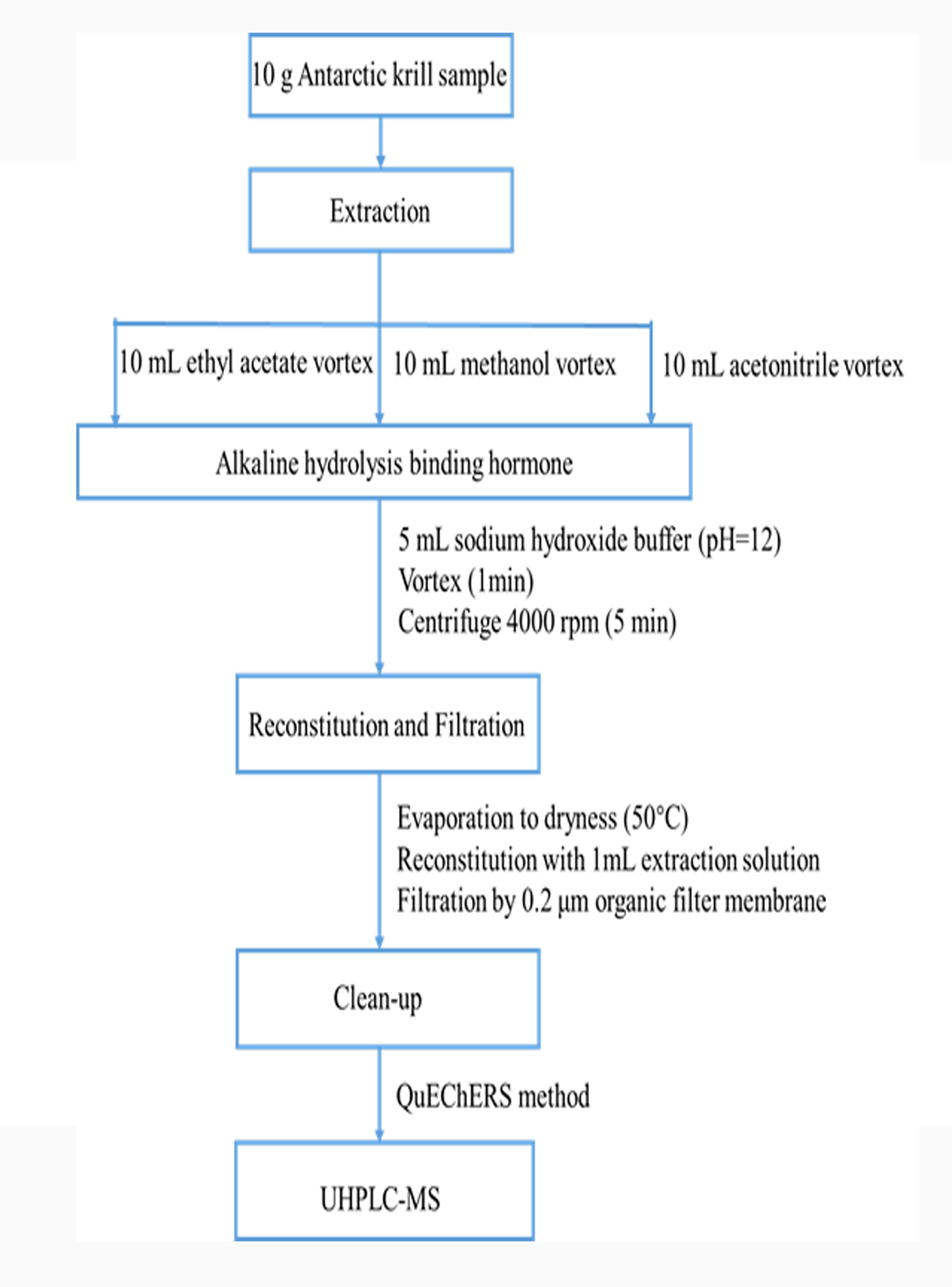

Supplement: S2 Fig — (TIF) [file pone.0213398.s002.tif]
