# Supplementary material for: Detection and analysis of 17 steroid hormones by ultra-high-performance liquid chromatography-electrospray ionization mass spectrometry (UHPLC-MS) in different sex and maturity stages of Antarctic krill (Euphausia superba Dana)
Source: PLoS One. 2019 Mar 11;14(3):e0213398. doi: 10.1371/journal.pone.0213398 (PMC6411355; doi:10.1371/journal.pone.0213398)
Supplement: S1 Table — (DOCX) [file pone.0213398.s003.docx]

**S1 Table. MS acquisition parameters at the ionization model of positive (ESI^+^) and negative (ESI^-^).**

|  |  |  |  |  |  |
| --- | --- | --- | --- | --- | --- |
| **Name** | **CAS** | **ESI** | **Present ion (m/z)** | **Product ion (m/z)** | **Collision energy (eV)** |
| Nandrolone | 434-22-0 | ESI+ | 275.1 | 109.0, 239.1 | 28,16 |
| Androsterone | 53-41-8 | ESI+ | 273.0 | 147.1,255.2 | 20,13 |
| Testosterone | 58-22-0 | ESI+ | 289.1 | 97.1,109.1 | 28,28 |
| Norethisterone | 68-22-4 | ESI+ | 299.2 | 109. 1,91. 1 | 28,42 |
| Testosterone Propionate | 57-85-2 | ESI+ | 345.5 | 97.1,109.1 | 29,29 |
| Progesterone | 57-83-0 | ESI+ | 315.5 | 97.1, 109.1 | 21,28 |
| Cortisol | 50-23-7 | ESI+ | 363.0 | 121.0,267.1 | 29,19 |
| Cortisone | 53-06-5 | ESI+ | 361.1 | 121.0,163.1 | 32,24 |
| Megestrol acetate | 595-33-5 | ESI+ | 385.0 | 224.2,287.2 | 27,18 |
| Prednisolone | 50-24-8 | ESI+ | 361.4 | 147.0,171.0 | 26,29 |
| Cortisone acetate | 50-04-4 | ESI+ | 403.3 | 163.2,343.3 | 26,19 |
| Dexamethasone | 50-02-2 | ESI+ | 393.0 | 361.2,375.3 | 17,13 |
| Aldosterone | 52-39-1 | ESI+ | 359.0 | 189.3,239.1 | 21,32 |
| Hydroxyprogesterone | 630-56-8 | ESI+ | 429.2 | 109.0,271.2 | 31,18 |
| Diethylstilbestrol | 56-53-1 | ESI- | 267.1 | 237.1,251.1 | 29,26 |
| Estradiol | 57-91-0 | ESI- | 271.1 | 145.1,183.1 | 53,43 |
| Estriol | 50-27-1 | ESI- | 287.1 | 143.1,171.1 | 56,40 |
|  |  |  |  |  |  |

Product ion:the underlined product ions were used for quantitative analysis.
