# Supplementary material for: Detection and analysis of 17 steroid hormones by ultra-high-performance liquid chromatography-electrospray ionization mass spectrometry (UHPLC-MS) in different sex and maturity stages of Antarctic krill (Euphausia superba Dana)
Source: PLoS One. 2019 Mar 11;14(3):e0213398. doi: 10.1371/journal.pone.0213398 (PMC6411355; doi:10.1371/journal.pone.0213398)
Supplement: S2 Table — The effects of hydrolysis, purification and extraction solvents. (DOCX) [file pone.0213398.s004.docx]

**S2 Table. The data of optimization.** The effects of hydrolysis, purification and extraction solvents.

|  |  |  |  |
| --- | --- | --- | --- |
|  | No-hydrolysis(ng) | Alkali-hydrolysis(ng) | Enzynamic-hydrolysis(ng) |
| Aldosterone | 23.1±1.5 | 32.93±2.1 | 32.97±1.8 |
| Testosterone | 9.57±0.8 | 10.43±1.2 | 10.6±1.5 |
| Progesterone | 44.3±4.2 | 56.3±3.8 | 51.2±5.1 |
| Estradiol | 31.47±6.2 | 36.17±4.2 | 36.33±3.1 |
| Estriol | 27.57±2.5 | 32.43±2.0 | 32.27±2.8 |
| Cortisol | 62.47±5.5 | 64.63±1.4 | 63.9±7.6 |
| Cortisone | 14.7±1.6 | 15.7±0.6 | 15.3±0.9 |
| Nandrolone | 12.97±1.5 | 16.53±2.6 | 15.2±2.1 |
| Megestrol acetate | 12.37±1.0 | 13.23±1.2 | 13.03±1.1 |
| Testosterone Propionate | 12.93±1.2 | 14.83±1.4 | 14.67±1.5 |
| Prednisolone | 3.5±0.4 | 3.83±0.6 | 3.84±0.8 |
| Hydroxyprogesterone | 38.23±2.1 | 39.97±2.6 | 39.63±1.8 |
| Cortisone acetate | 294.8±19.1 | 334.3±15.4 | 331.7±8.9 |
| Dexamethasone | 154.5±12.5 | 167.1±13.1 | 158.3±11.4 |
|  |  |  |  |

**Continued：**

|  |  |  |  |
| --- | --- | --- | --- |
|  | HLB(ng) | C_18_(ng) | QuECHERS(ng) |
| Aldosterone | 36.73±4.6 | 31.23±3.8 | 37.17±4.8 |
| Testosterone | 7.37±2.1 | 7.07±1.8 | 10.37±1.4 |
| Progesterone | 56.5±2.9 | 52.2±3.4 | 56.4±1.5 |
| Estradiol | 39±2.1 | 38.47±1.9 | 39.27±3.0 |
| Estriol | 28.64±0.9 | 27.37±1.8 | 28.53±1.2 |
| Cortisol | 62.47±3.5 | 63.1±2.2 | 64.1±2.6 |
| Cortisone | 16.4±1.4 | 15.87±0.8 | 16.97±0.6 |
| Nandrolone | 16.67±2.3 | 16.3±1.1 | 16.63±1.3 |
| Megestrol acetate | 13.9±2.5 | 12.84±1.6 | 14.23±1.4 |
| Testosterone Propionate | 12.67±1.0 | 12.46±0.9 | 14.3±1.5 |
| Prednisolone | 3.63±0.9 | 3.57±0.6 | 3.8±0.8 |
| Hydroxyprogesterone | 26.57±2.2 | 26.43±1.8 | 26.89±2.1 |
| Cortisone acetate | 325.83±12.4 | 325.3±8.6 | 330.97±11.7 |
| Dexamethasone | 156.83±8.6 | 155.83±5.9 | 161.87±6.4 |
|  |  |  |  |

**Continued:**

|  |  |  |  |
| --- | --- | --- | --- |
|  | Ethyl acetate(ng) | Methanol(ng) | Acetonitrile(ng) |
| Aldosterone | 38.14±2.4 | 29.5±3.1 | 23.63±1.6 |
| Testosterone | 12.77±1.2 | 10.47±0.8 | 11.5±1.4 |
| Progesterone | 57.74±2.8 | 68.53±3.1 | 48.27±3.3 |
| Estradiol | 39.37±2.4 | 32.5±1.8 | 38.3±2.1 |
| Estriol | 31.4±1.8 | 34.5±2.2 | 31.87±1.9 |
| Cortisol | 64.3±2.3 | 68.14±4.1 | 62.3±2.0 |
| Cortisone | 17.8±1.4 | 17.47±1.9 | 16.33±1.3 |
| Nandrolone | 16.87±1.8 | 15.87±1.6 | 12.94±1.7 |
| Megestrol acetate | 18.5±1.5 | 15.27±1.6 | 12.5±0.9 |
| Testosterone Propionate | 12.33±1.1 | 12.27±0.9 | 11.74±1.7 |
| Prednisolone | 3.83±0.7 | 10.07±1.6 | 21.54±0.8 |
| Hydroxyprogesterone | 29.67±2.1 | 28.6±1.6 | 29.37±0.8 |
| Cortisone acetate | 325.1±12.4 | 316±18.2 | 309.9±10.9 |
| Dexamethasone | 163.13±6.6 | 162.9±8.7 | 157.54±6.5 |
|  |  |  |  |
