# Supplementary material for: Detection and analysis of 17 steroid hormones by ultra-high-performance liquid chromatography-electrospray ionization mass spectrometry (UHPLC-MS) in different sex and maturity stages of Antarctic krill (Euphausia superba Dana)
Source: PLoS One. 2019 Mar 11;14(3):e0213398. doi: 10.1371/journal.pone.0213398 (PMC6411355; doi:10.1371/journal.pone.0213398)
Supplement: S3 Table — (DOCX) [file pone.0213398.s005.docx]

**S3 Table.** Detected hormones and their contents in Antarctic krill.

|  |  |
| --- | --- |
|  | Mean ± SD (ng/g) |
| Aldosterone | 381.4±9.8 |
| Testosterone | 127.7±11.4 |
| Progesterone | 685.3±14.5 |
| Estradiol | 393.7±12.1 |
| Estriol | 646.3±15.2 |
| Cortisol | 178±8.4 |
| Cortisone | 168.7±11.6 |
| Nandrolone | 185±11.5 |
| Megestrol acetate | 142.3±7.3 |
| Testosterone Propionate | 215.4±12.2 |
| Prednisolone | 396.3±10.9 |
| Hydroxyprogesterone | 3343.2±29.6 |
| Cortisone acetate | 1631.3±17.8 |
| Dexamethasone | 345±14.8 |
|  |  |
