# Supplementary material for: Detection and analysis of 17 steroid hormones by ultra-high-performance liquid chromatography-electrospray ionization mass spectrometry (UHPLC-MS) in different sex and maturity stages of Antarctic krill (Euphausia superba Dana)
Source: PLoS One. 2019 Mar 11;14(3):e0213398. doi: 10.1371/journal.pone.0213398 (PMC6411355; doi:10.1371/journal.pone.0213398)
Supplement: S4 Table — (DOCX) [file pone.0213398.s006.docx]

**S4 Table.** **The changes of endogenous steroid hormones and exogenous steroid hormones.**

|  |  |  |  |  |  |
| --- | --- | --- | --- | --- | --- |
|  | Aldosterone(ng/g) | Testosterone propionate(ng/g) | Estriol(ng/g) | Megestrol acetate(ng/g) | Cortisone acetate(ng/g) |
| 1＋ | 136.84±5.4 | 129.16±2.6 | 0 | 82.5±1.4 | 2481.16±7.4 |
| 2＋ | 157.62±6.2 | 153.73±3.8 | 47.08±0.4 | 34.43±0.6 | 3197.49±11.6 |
| 3＋（♀） | 254.74±4.8 | 154.24±1.2 | 295.95±3.4 | 33.14±0.8 | 5406.22±14.3 |
| 3＋（♂） | 258.14±8.2 | 147.49±2.3 | 56.09±1.1 | 32.76±0.7 | 5299.72±13.2 |
| 4＋（♀） | 248.78±4.9 | 151.18±2.4 | 539.13±4.5 | 37.38±0.9 | 1494.47±6.4 |
| 4＋（♂） | 330.86±6.5 | 145.2±2.2 | 190.86±2.6 | 42.76±0.6 | 3032.82±10.9 |
| 5＋（♀） | 146.23±4.5 | 136.58±1.1 | 686.45±5.1 | 156.69±4.9 | 2235.78±8.4 |
| 5＋（♂） | 193.87±4.7 | 143.15±2.7 | 230.75±2.2 | 47.76±1.1 | 2998.75±9.2 |
|  |  |  |  |  |  |

**Continued:**

|  |  |  |  |  |  |
| --- | --- | --- | --- | --- | --- |
|  | Dexamethasone(ng/g) | Hydroxyprogesterone(ng/g) | Cortisone(ng/g) | Nandrolone(ng/g) | Prednisolone(ng/g) |
| 1＋ | 1419.75±10.7 | 20.02±0.6 | 138.86±3.4 | 335.63±6.2 | 104.48±3.1 |
| 2＋ | 605.31±6.2 | 161.51±2.4 | 110.49±2.6 | 196.31±4.1 | 33.32±0.4 |
| 3＋（♀） | 2050.76±26.4 | 235.54±5.4 | 222.4±4.2 | 163.47±4.4 | 34.87±0.6 |
| 3＋（♂） | 594.86±5.4 | 401.1±8.4 | 188.91±3.7 | 258.23±4.8 | 45.39±0.8 |
| 4＋（♀） | 1538.95±10.9 | 350.04±6.8 | 148.58±3.8 | 151.12±3.9 | 232.5±5.3 |
| 4＋（♂） | 759.53±7.8 | 488.08±9.2 | 139.24±3.2 | 170.37±4.2 | 161.07±5.3 |
| 5＋（♀） | 2694.28±26.2 | 387.87±7.4 | 139.94±2.9 | 144.59±3.8 | 208.5±3.8 |
| 5＋（♂） | 309.63±4.9 | 154.4±3.2 | 93.3±2.4 | 240.59±4.4 | 127.9±4.4 |

**Continued:**

|  |  |  |  |  |
| --- | --- | --- | --- | --- |
|  | Cortisol(ng/g) | Progesterone(ng/g) | Estradiol(ng/g) | Testosterone(ng/g) |
| 1＋ | 76.85±0.9 | 0 | 0 | 0 |
| 2＋ | 717.22±4.2 | 0 | 68.89±0.8 | 31.22±0.8 |
| 3＋（♀） | 724.86±3.9 | 328.97±4.9 | 521.33±4.8 | 64.59±1.2 |
| 3＋（♂） | 611.54±4.3 | 86.68±1.1 | 197.13±2.4 | 91.18±1.4 |
| 4＋（♀） | 403.99±3.8 | 636.24±5.4 | 647.52±5.6 | 84.61±0.8 |
| 4＋（♂） | 600.47±4.1 | 141.25±5.2 | 107.31±1.7 | 98.14±1.1 |
| 5＋（♀） | 536.16±4.5 | 628.73±6.3 | 473.16±5.1 | 36.98±0.6 |
| 5＋（♂） | 394.12±3.5 | 129.55±2.4 | 90.32±0.9 | 75.42±1.0 |
|  |  |  |  |  |
